# Intra-population variability of the saccular, utricular and lagenar otoliths of the garfish *Belone belone* (Linnaeus, 1760) from South-Western Ionian Sea (Central Mediterranean Sea)

**DOI:** 10.1186/s12862-024-02219-0

**Published:** 2024-03-11

**Authors:** Claudio D’Iglio, Marco Albano, Sergio Famulari, Alex Carnevale, Serena Savoca, Nunziacarla Spanò, Gioele Capillo

**Affiliations:** 1 Department of Chemical, Biological, Pharmaceutical and Environmental Sciences, University of Messina, Viale Ferdinando Stagno d’Alcontres 31, 98166 Messina, Italy; 2 Department of Veterinary Sciences, University of Messina, Polo Universitario Dell’Annunziata, 98168 Messina, Italy

**Keywords:** Garfish, *Belone belone*, Ionian Sea, *Sagittae*, *Lapilli*, *Asterisci*, Shape analysis

## Abstract

The garfish *Belone belone* represents the only valid endemic *Belone* species for the Mediterranean Sea and the eastern Atlantic Ocean. It shows a wide global distribution range, with a high commercial value and ecological relevance in the pelagic domain. Despite this, there needs to be more knowledge regarding the otoliths of this species, with the total absence of descriptions regarding *asterisci* and *lapilli* from Mediterranean populations and a lack of studies on the reliability of shape analysis on its *sagittae.* The present paper aims to provide the first main contours description of the three otoliths pairs from a Mediterranean population, providing an accurate investigation of morphology, morphometry, and intra-specific variability of *sagittae, lapilli,* and *asterisci*. Results showed (i) the absence of directional bilateral asymmetry and sexual asymmetry for the three otoliths pairs, (ii) a different morphology and morphometry of *sagittae, lapilli* and *asterisci* than those described in the literature, and (iii) an enhanced variability between *sagittae* morphometry and shape between the three investigated size classes. All these data confirmed the reliability of the studied species of shape analysis, showing a geographical and size-related variability of otoliths features probably related to genetics, environmental conditions, and life habits variations.

**Supplementary Information:**

The online version contains supplementary material available at 10.1186/s12862-024-02219-0.

## Background

The family Belonidae, order Beloniformes, includes ten genera and 34 species of freshwater and marine teleost known as needlefishes [1, 2]. They are characterized by an elongated body and long upper and lower jaws, resulting in a beak with a large mouth opening equipped with sharp needle-like teeth. Small cycloid scales are distributed along their lateral lines. A separation in the third pair of the upper pharyngeal bones is typical of this family, together with the absence of spines in the fins, no finlets behind the anal and the dorsal fins, and the nostrils placed in a pit anteriorly to the eyes [3, 4]. All needlefishes’ species are oviparous and live close to the surface. They are ichthyophage predators, hunting on small fishes using their beaks. In the Mediterranean Sea, they have been recognized six species of needlefishes belonging to four genera: *Ablennes hians*, Valenciennes, 1846, *Belone belone,* Linnaeus, 1761, *Belone svetovidovi*, Collette & Parin, 1970, *Tylosurus acus acus*, Lacépède, 1803, *Tylosurus acus imperialis*, Rafinesque, 1810, and the non-indigenous indo-pacific species *Tylosurus choram*, Rüppell, 1837 [4–6].

Concerning *B. belone*, in 1970 [7], they were acknowledged three subspecies according to their global distribution (*B. b. belone*, Linnaeus, 1761, *B. b. euxini*, Günther, 1866, *B. b. gracilis*, Lowe, 1839), but recently the garfish *B. belone* was accepted as the only valid endemic species for the Mediterranean Sea and the eastern Atlantic Ocean [8]. This species shows a wide distribution range, inhabiting brackish and marine environments from Norway to the Canaries, in addition to the Mediterranean and Black Seas. Like other pelagic teleosts, it is an oceanodromous species. It inhabits the offshore areas, moving near the coast during spawning season. It is during this migratory pattern that garfish populations are more susceptible to fisheries activities [9, 10], being mainly caught using floating gill nets and pelagic trawling. In the Adriatic Sea and Turkish Mediterranean waters, garfish is the principal target species of seine nets, representing also a by-catch species in purse seine fisheries [3, 11–13]. In the Mediterranean Sea, the *B. belone* capture production has been growing since 2016, stationing at 621 tons in 2018, with Turkey, Tunisia, Greece, and Spain as the leading countries for its harvesting and consumption [14]. This species is among the most important pelagic commercial species of Turkey, especially for the Black Sea’s Turkish artisanal fisheries and Tunisia, representing the main belonids species for catch in the entire Mediterranean basin [15–17]. In addition to its commercial value, garfish plays a fundamental ecological role in the pelagic domain. It is an opportunistic predator that switches its prey preferences from crustaceans (e.g., copepods, decapods larvae, amphipods) to teleost fishes (e.g., clupeids, engraulids, horse mackerels), according to its size and the preys’ availability [18–21]. Additionally, it is among the principal preys of larger pelagic fishes and marine mammals [22–24]. Due to its high commercial and ecological value, many studies have been performed on the biology of this species. These provided essential information for accurate stock assessments, exploring its population dynamics (e.g., age structure, growth, mortality, reproductive cycle), metazoan parasites communities, and trophic ecology in the different Mediterranean geographical areas [12, 15, 17, 21, 25–30]. However, relatively few researches have been performed on garfish otoliths, another fundamental tool for stock assessment and population studies [31–36].

Otoliths are paired carbonate structures in the vertebrates’ inner ears, close to the midbrain, with auditory and vestibular functions [37]. Teleost’s inner ear (one for each side) is characterized by a great morphological variability among different taxa [38]. Its basic structure, common in bony and cartilaginous fishes, is characterized by three semicircular canals, with their end organs (*ampullae*) and three otoliths end organs (*saccule, utricle, lagena)*. Otoliths are located inside these lasts (one otolith in each organ: respectively, *sagitta, lapillus,* and *asteriscus*), which, according to their taxon-specific orientation related to the fish’s rostrocaudal axis, are essential for the localization of sound sources [39]*.* They are composed of calcium carbonate and non-collagenous organic matrix, depositing daily for the entire fishes’ lifetime [40, 41], and, among the three otoliths pairs, *sagittae* have long been the most studied. Due to their species-specific morphology, timekeeping and chemical properties, and variability at intra and interspecific levels, *sagittae* long been widely used in many research fields, including taxonomy and paleoethology, to trophic ecology and fisheries science [33, 42–53]. Data on *lapilli* and *asterisci* are very few and fragmentary, especially concerning the marine teleost species; this is attributable to their dimension*,* smaller than *sagittae* in non-ostariophysian fishes [54–56], and, especially for *asterisci,* mainly composed of vaterite*,* to their low resistance to the extraction process. It has also been claimed that *lapilli* and *asterisci* show a low intra and inter-specific variability, considerably less evident than *sagittae* [57, 58]. Conversely, recent findings by several authors have assessed, also in otophysans species, a substantial inter-specific diversity valuable for species identification and evident intra-specific variations between different populations related to environmental factors, as also confirmed in not-otophysans species [57–61]. Indeed, according to T. Schulz-Mirbach et al. [59], providing new information on *lapilli* and *asterisci* of the different teleost’s species, also applying shape analysis, is required to evaluate the additional data provided by the investigations on all the otoliths pairs, improving species identification processes, stock assessment, and fisheries management.

Concerning *B. belone* otoliths, literature data are scarce, with few studies on *sagittae,* describing their gross morphology, the relations between fish length and otolith size, and some morphometrical features [62–66]. Only three studies describe *lapilli* and *asterisci* in populations inhabiting the Mediterranean Sea and Portuguese Atlantic waters [57, 58, 67]. In this context, the present paper aims to analyze the three otoliths pairs of garfish from the Ionian Sea (the Italian Sea with the highest values of capture production for this species in 2020 [68]) through morphological, morphometrical, and shape analysis, in order to investigate the intra specific variability of *sagittae, lapilli* and *asterisci* between different size classes, otoliths pairs and populations, comparing data from present paper to those from literature. The present paper can allow (i) to investigate the presence of otoliths’ bilateral asymmetry and sexual variability inside the analyzed population, (ii) to compare otoliths’ morphology and morphometry between the Ionian Sea and other geographical areas through a comparison with literature data and (iii) to give an accurate otoliths’ contours description, essential for stock assessment.

All this information is fundamental to improving the knowledge base on the eco-morphological adaptation of this species to the pelagic environment, filling the gap in the morphology, shape, and intra-specific variability of *sagittae, lapilli,* and *asterisci.* Indeed, there needs to be more data on *sagittae* from the Ionian Sea and an almost total absence of studies on *lapilli* and *asterisci,* especially from the Mediterranean Sea, regarding the studied species [57, 58, 67]. Moreover, taking into account the difficulties in assessing the stock composition and discriminating among populations of medium-large size pelagic fishes due to their broad geographical range and the absence of biogeographical barriers [69, 70], the present paper can improve the capability to distinguish separated fish groups of *B. belone,* providing the first accurate shape analysis on the three otoliths pairs. This is fundamental to correctly distinguishing populations and stocks for better conservation and improved fisheries management.

## Materials and methods

### Sample collection, processing, and image analysis

**Fig. 1:**
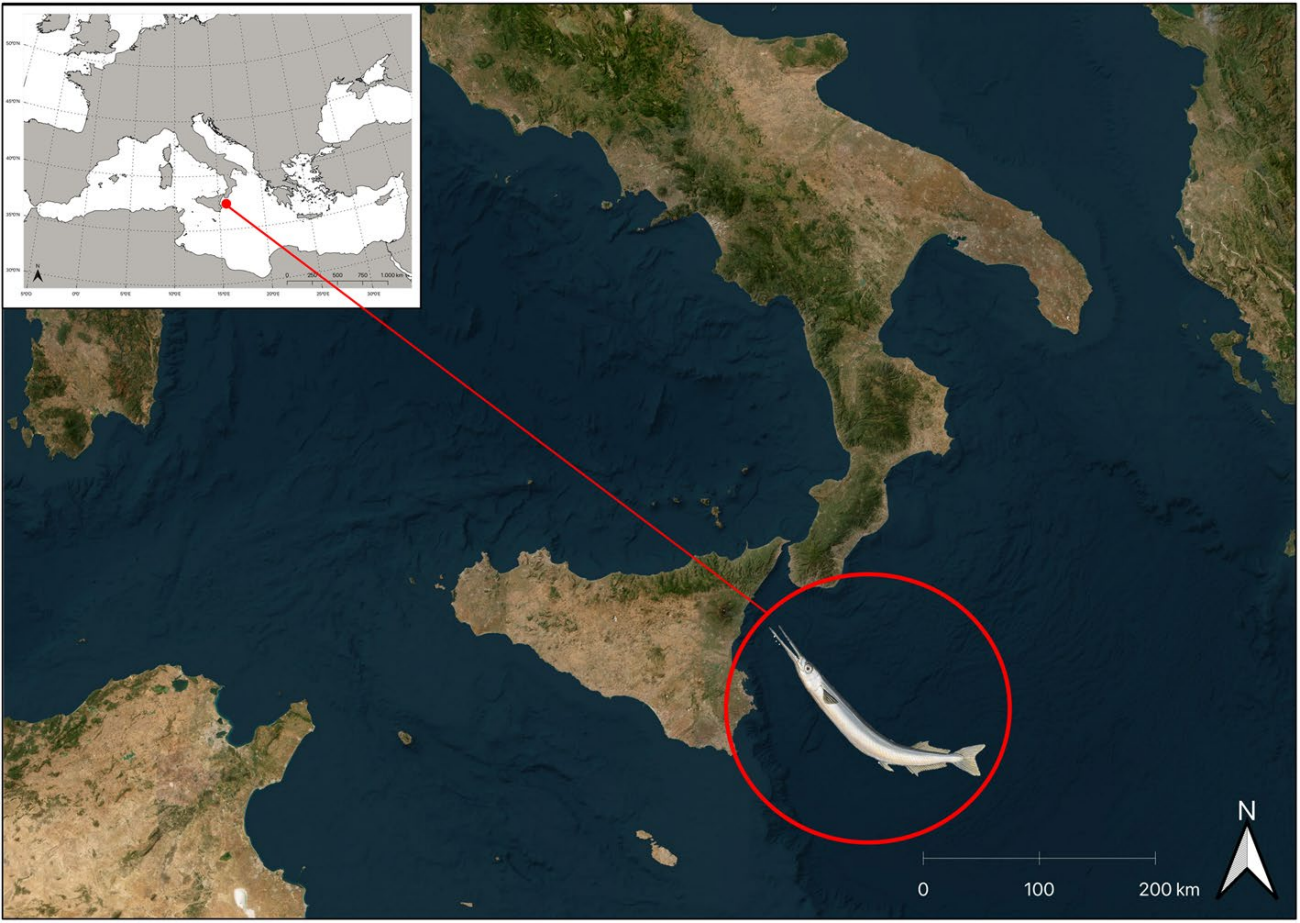
Map of the Mediterranean Sea with sampling area of *B. belone* specimens analyzed in this study highlighted by the red circle

Sixty-five specimens of *B. belone* were obtained already dead from a market in Catania (Italy, Sicily) supplied by local artisanal fisheries operating in the Ionian Sea (FAO statistical division 37.2.2-Ionian Sea; Fig. 1).

Samples were transported still frozen in the laboratory, where each specimen was weighted (Total Weight: TW, g), measured (Total Length: TL, mm) and sexed. Individuals were assigned to three size classes according to its TL (Class I: TL ≤ 240 mm; Class II: 240 mm < TL ≤ 290 mm; Class III: TL > 290 mm), using K-means clustering method. Each otolith (*sagitta, lapillus,* and *asteriscus* of each inner ear) was extracted and gently washed in 3% H_2_O_2_ and Milli-Q water to remove tissue remains. Once dried, each *sagitta, lapillus,* and *asteriscus* face was photographed twice using the Axiocam 208 color camera (Carl Zeiss, Jena, Germany) under a stereomicroscope Zeiss Discovery V8. Otolith images were processed and converted into binary format for shape analysis using ImageJ 1.48p software [71]. Once obtained from shape analysis, data on maximum otolith length, OL mm, maximum otolith width, OW mm, otolith perimeter, OP mm and otolith surface, OS, mm^2^ for each analyzed otolith, investigated the relation between otolith and fish length calculating the ratio of otolith length to the total fish length (OL/TL). Moreover, in order to evaluate how otoliths’ morphometric relationships and morphology features change intra-specifically, they were calculated several shape indices, according to literature [72–77]: circularity (C = OP^2^/OS), rectangularity (Re = OS/[OL × OW]), ellipticity (E = OL–OW/OL + OW), aspect ratio (AR = OW/OL%), form factor (FF = 4πOS/OP^2^) and roundness (Ro = 4OS/πOL^2^).

### Shape analysis

The otoliths outlines were used to perform the Shape analysis using the open-access package shape R on R software (RStudio 2022.07.1 Build 554; R Gui 4.1.3 2022.03.10). This package was designed for the otolith shape studies, widely used to analyze the intra and inter-specific variations in teleost species and populations [78]. ImageJ software (version 1.53 k freely available at https://imagej.nih.gov/ij/) was used to binarize each *sagitta, lapilli* and *asterisci* picture, which, subsequently, was classified according to fish size classes, sex, and otoliths’ side. The greyscale threshold was set at 0.05 (intensity threshold) to detect the outlines using a specific shape R function. The data file with analyzed specimens’ information (e.g., body weight and fish length) was linked to the extracted contours. The getMeasurements function was applied to calculate the otoliths’ length, width, area, and perimeter for each specimen on the previously detected outlines. The allometric relationships were assessed between fish lengths and otolith shapes, extracting Wavelet and Fourier coefficients, adequately adjusted, through proper functions of Shape R. The intra-specific comparison between the mean otolith shapes was obtained using the Wavelet coefficients, estimating the quality of the reconstruction through the analysis of the deviation of the coefficients’ reconstruction from the otolith outline (Fig. S1). A specific function of the g-plots R package was applied to investigate the influence of the position along the outline on the wavelet coefficients’ variation (Fig. S2).

### Data analysis

Univariate and multivariate statistical methods were applied to conduct investigations on *asterisci*, *lapilli,* and *sagittae* using Prism V.8.2.1 (Graph-pad Software Ltd., La Jolla, CA 92037, USA), R vegan package V.2.5, and PAST V.4.

An unpaired t-test was used as a tool to investigate the occurrence of differences in morphometric parameters between sexes and between right and left otoliths. Any otolith morphometric variations between the different size classes investigated were detected using a one-way analysis of variance (one-way ANOVA) and Linear Discriminant Analysis (LDA). Additionally, the correlation between the measured parameters and fish body weight (BW) and total length (TL) was tested using the Pearson correlation coefficient.

To explore the variation of otolith contours between specimens, the shape indices were extrapolated and analyzed through an ANOVA-like permutation test and a Linear Discriminant Analysis (LDA) to obtain an overview of the differences in otolith shape between right and left side, gender, and size classes examined. The significance level of the *p*-value was set at < 0.05.

## Results

A total of 65 individuals (20 males and 45 females) were examined, with 18 specimens classified as Class I, 35 as Class II, and 12 as Class III.

### Morphometric and Shape analysis of Sagittae

**Fig. 2:**
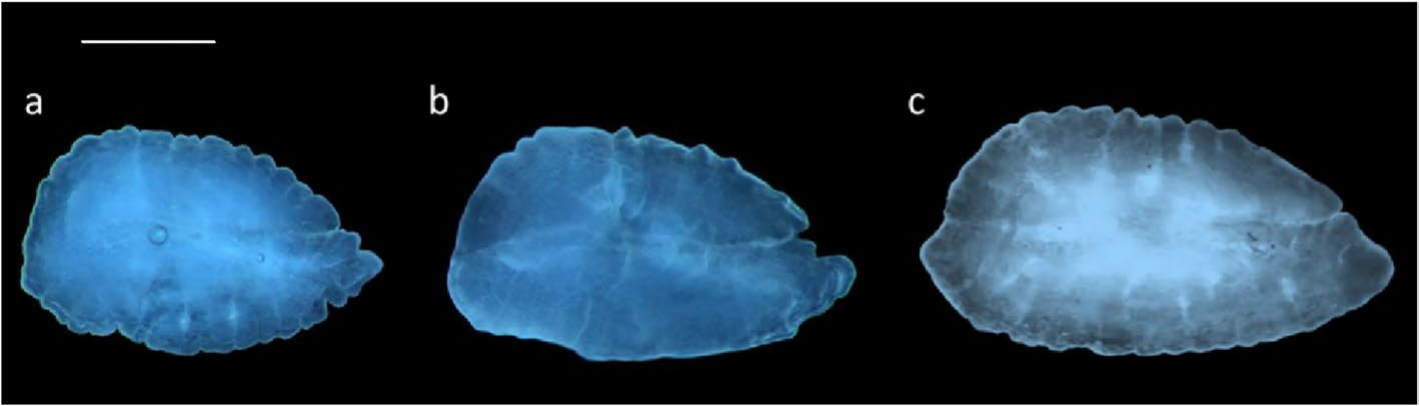
Stereoscope images of left *sagittae* belonging to size class I (**a**), II (**b**) and III (**c**). Scale bar: 1 cm

According to the terminology proposed by Tuset et al., Nolf and Assis [66, 67, 79], *B. belone* showed overall elliptic-lanceolate *sagittae* (Fig. 2 a-c), characterized by peaked anterior region, with a short *rostrum*, pointed and broad, and a round posterior region. The *antirostrum* was generally absent, except in some specimens, in which it was very short, broad, and pointed. The *excisura ostii* was absent or wide, changing in the different individuals according to the size class of belonging.

In Table 1 they were reported the morphometric parameters assessed for the studied specimens.

**Table 1:** *Sagittae* morphometric mean values, with standard deviation (s.d.), maximum (Max.), and minimum (Min.) values, for each investigated size class: maximum otolith length (OL, mm) and otolith width (OW, mm), otolith perimeter (OP, mm), otolith surface (OS, mm^2^), otolith length to the total fish length ratio (OL/TL), circularity (C), rectangularity (Re), ellipticity (E), aspect ratio (AR), form factor (FF) and roundness (Ro)

	**Class I**			**Class II**			**Class III**		
	**Mean**	**s.d**	**Min—Max**	**Mean**	**s.d**	**Min—Max**	**Mean**	**s.d**	**Min—Max**
**OL**	2.16	0.23	1.7—2.67	2.36	0.21	2.02—2.88	2.64	0.27	2.05—3.33
**OW**	1.29	0.13	1.01—1.55	1.37	0.1	1.64—1.15	1.54	0.11	1.31—1.79
**OP**	6.46	0.71	4.93—7.88	7	0.57	5.85—8.25	7.8	0.74	6.3—9.34
**OS**	2.08	0.41	1.29—2.97	2.34	0.35	1.75—3.28	2.97	0.45	2.12—4.1
**OL/TL %**	0.95	0.1	0.79—1.21	0.9	0.08	0.71—1.09	0.82	0.11	0.67—1.11
**C**	20.28	1.5	17.65—24.75	21.02	1.26	18.86—23.7	20.6	1.28	18.58—23.37
**Re**	0.74	0.02	0.7—0.79	0.72	0.02	0.67—0.79	0.73	0.02	0.69—0.77
**E**	0.25	0.03	0.19—0.33	0.26	0.03	0.19—0.32	0.26	0.03	0.2—0.32
**AR**	0.6	0.04	0.5—0.69	0.58	0.03	0.51—0.68	0.58	0.04	0.52—0.66
**FF**	0.62	0.04	0.51—0.71	0.6	0.03	0.53—0.66	0.61	0.04	0.54—0.67
**Ro**	0.56	0.05	0.47—0.68	0.54	0.04	0.47—0.64	0.54	0.04	0.47—0.64

The examination showed no significant variation between the right and left *sagittae* (*p* > 0.05).

Gender represented a discriminating factor only for 3 of the morphometric parameters investigated, namely OS (*p* = 0.014), OL (*p* = 0.025) and E (*p* = 0.01).

**Fig. 3:**
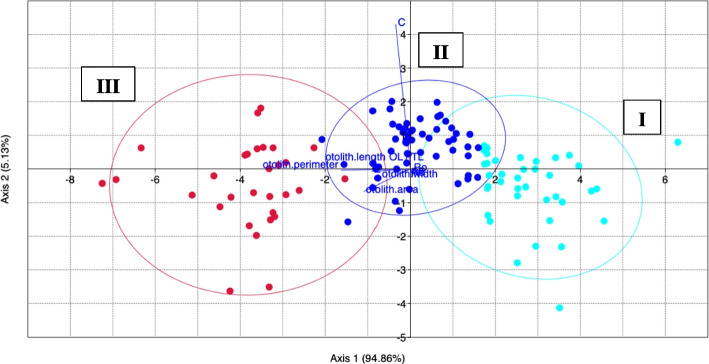
Linear Discriminant Analysis (LDA) between *sagittae* morphometric parameters from the three investigated size classes (aqua dots I, blue dots II and red dots III)

Finally, the size class strongly influenced the morphometric variability observed between the samples, as confirmed by LDA (Fig. 3). Results, reported in Table 2, highlighted how AR was the only parameter that did not vary between size classes (*p* > 0.05).

**Table 2:** Results of ANOVA carried out between biometric data (total fish length, TL, and body weight, BW) morphometric sagittal parameters of the investigated specimens belonging to the three size classes, with significant results set at *p* < 0.05: maximum otolith length (OL, mm) and otolith width (OW, mm), otolith perimeter (OP, mm), otolith surface (OS, mm^2^), otolith length to the total fish length ratio (OL/TL), circularity (C), rectangularity (Re), ellipticity (E), aspect ratio (AR), form factor (FF) and roundness (Ro)

Tukey's multiple comparisons test	95,00% CI of diff,	Summary	*P* Value
TL_mm I vs. TL _mm II	-42,88 to -26,68	****	< 0.0001
TL _mm I vs. TL _mm III	-106,9 to -88,19	****	< 0.0001
TL _mm II vs. TL_mm III	-71,50 to -54,07	****	< 0.0001
BW_g I vs. BW_g II	-1036 to 57,77	ns	0.0896
BW_g I vs. BW_g III	-2252 to -986,3	****	< 0.0001
BW_g II vs. BW_g III	-1718 to -542,1	****	< 0.0001
OS I vs. OS II	-0,4661 to -0,07006	**	0.0048
OS I vs. OS III	-1,122 to -0,6634	****	< 0.0001
OS II vs. OS III	-0,8377 to -0,4115	****	< 0.0001
OL I vs. OL II	-0,3109 to -0,07977	***	0.0003
OL I vs. OL III	-0,6152 to -0,3476	****	< 0.0001
OL II vs. OL III	-0,4104 to -0,1618	****	< 0.0001
OW I vs. OW II	-0,1401 to -0,02504	**	0.0026
OW I vs. OW III	-0,3183 to -0,1852	****	< 0.0001
OW II vs. OW III	-0,2311 to -0,1073	****	< 0.0001
OP I vs. OP II	-0,8692 to -0,2118	***	0.0005
OP I vs. OP III	-1,723 to -0,9623	****	< 0.0001
OP II vs. OP III	-1,156 to -0,4487	****	< 0.0001
OL / TL I vs. OL / TL II	5,377e-005 to 0,0009866	*	0.0248
OL / TL I vs. OL / TL III	0,0007877 to 0,001868	****	< 0.0001
OL / TL II vs. OL / TL III	0,0003057 to 0,001309	***	0.0006
C I vs. C II	-1,417 to -0,07310	*	0.0259
C I vs. C III	-1,103 to 0,4529	ns	0.5838
C II vs. C III	-0,3028 to 1,143	ns	0.3552
Re I vs. Re II	0,006217 to 0,02819	***	0.0009
Re I vs. Re III	0,001084 to 0,02653	*	0.03
Re II vs. Re III	-0,01522 to 0,008423	ns	0.7742
E I vs. E II	-0,02559 to 0,003630	ns	0,1795
E I vs. E III	-0,02631 to 0,007517	ns	0,3877
E II vs. E III	-0,01414 to 0,01730	ns	0,9691
AR I vs. AR II	-0,004328 to 0,03268	ns	0.1681
AR I vs. AR III	-0,009358 to 0,03349	ns	0.3778
AR II vs. AR III	-0,02202 to 0,01780	ns	0.9657
FF I vs. FF II	0,003775 to 0,04212	*	0.0145
FF I vs. FF III	-0,01156 to 0,03283	ns	0.493
FF II vs. FF III	-0,03294 to 0,008320	ns	0.3359
Ro I vs. Ro II	0,003644 to 0,04910	*	0.0185
Ro I vs. Ro III	-0,004466 to 0,04816	ns	0.124
Ro II vs. Ro III	-0,02898 to 0,01993	ns	0.8993

Some morphometric parameters showed a strong positive correlation with the biometric data of the examined specimens (total length, TL, and body weight, BW), except for the OL/TL variable, which, on the contrary, exhibited a negative correlation with the body weight and the total length of the examined species. The Pearson correlation results are shown in Table 3.

**Table 3:** Pearson correlation results between biometric data (total fish length, TL, and body weight, BW) of the investigated species and morphometric sagittal parameters

	**Pearson Correlation**			
	r	95% confidence interval	*P* value	*P* value summary
TL (mm) vs OS	0.6358	0.5169 to 0.7306	< 0.0001	****
TL (mm) vs OL	0.5933	0.4651 to 0.6971	< 0.0001	****
TL (mm) vs OW	0.6313	0.5114 to 0.7271	< 0.0001	****
TL (mm) vs OP	0.5874	0.4580 to 0.6925	< 0.0001	****
TL (mm) vs OL / TL	-0.5716	-0.6798 to -0.4390	< 0.0001	****
TL (mm) vs C	0.03367	-0.1442 to 0.2095	0.7116	ns
TL (mm) vs Re	-0.1669	-0.3341 to 0.01043	0.065	ns
TL (mm) vs E	0.0798	-0.0986 to 0.2533	0.380	ns
TL (mm) vs AR	-0.08159	-0.2549 to 0.09684	0.3696	ns
TL (mm) vs FF	-0.04425	-0.2196 to 0.1338	0.627	ns
TL (mm) vs Ro	-0.1244	-0.2950 to 0.05380	0.1703	ns
BW (g) vs OS	0.3184	0.1498 to 0.4690	0.0003	***
BW (g) vs OL	0.2752	0.1032 to 0.4312	0.0021	**
BW (g) vs OW	0.3428	0.1765 to 0.4901	0.0001	***
BW (g) vs OP	0.26	0.08697 to 0.4178	0.0037	**
BW (g) vs OL / TL	-0.378	-0.5203 to -0.2154	< 0.0001	****
BW (g) vs C	-0.1007	-0.2729 to 0.07770	0.2677	ns
BW (g) vs Re	-0.07778	-0.2513 to 0.1006	0.3925	ns
BW (g) vs E	-0.0255	-0.2017 to 0.1522	0.7789	ns
BW (g) vs AR	0.02288	-0.1548 to 0.1991	0.8017	ns
BW (g) vs FF	0.08955	-0.08890 to 0.2624	0.3246	ns
BW (g) vs Ro	-0.01331	-0.1899 to 0.1641	0.8838	ns

**Fig. 4:**
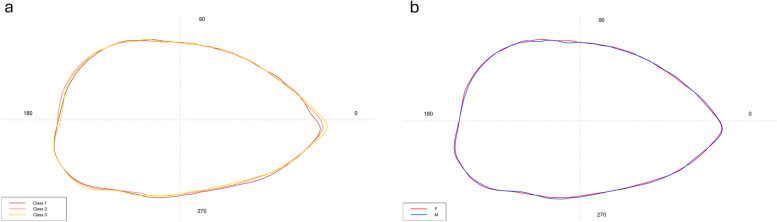
Mean shape of the left sagittae contours belonging to the three size classes (**a**) and to males (M) and females (F) (**b**)

As reported in Fig. 4a, the shape analysis showed a marked *excisura ostii* in *sagittae* belonging to Class I, with regular posterior, slightly lobed margins. Dorsal and ventral margins were crenate, with sculptures that become evident in size classes II and III. Specimens belonging to these last size classes showed a most enhanced *rostrum*, and a most marked irregularity of the margins, then the first. Concerning the differences between male and female specimens (Fig. 4b), the mean contours evidenced only a slight variation in margin crenation, more marked in males than females, especially in the dorsal margins.

**Fig. 5:**
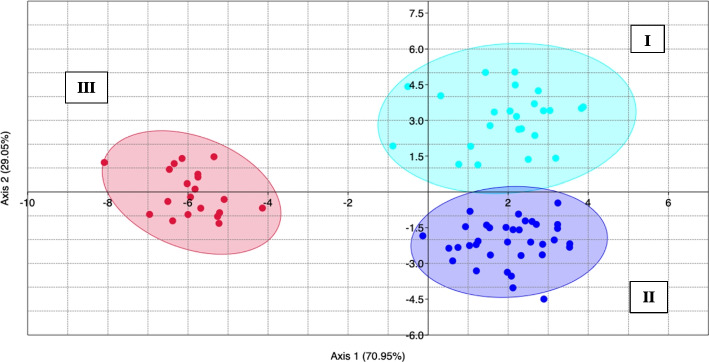
Linear Discriminant Analysis (LDA) between elliptic Fourier descriptors calculated for the different size classes (aqua dots I, blue dots II and red dots III). Ellipses include a 95% confidence interval

Given the low significance of the differences observed between the right and left sides, these data were excluded from subsequent analyses. ANOVA and LDA analyzed the wavelet coefficients obtained by the shape analysis to provide an overview of the diversity of sagittal contours between specimens of the opposite sex and between the size classes investigated in the present study. ANOVA showed significant differences between sagittal contours of different size classes (*p* < 0.05), as confirmed by LDA (Fig. 5). Shape indexes did not show significant variation between sexes (*p* < 0.05).

### Morphometric and Shape analysis of Lapilli

**Fig. 6:**
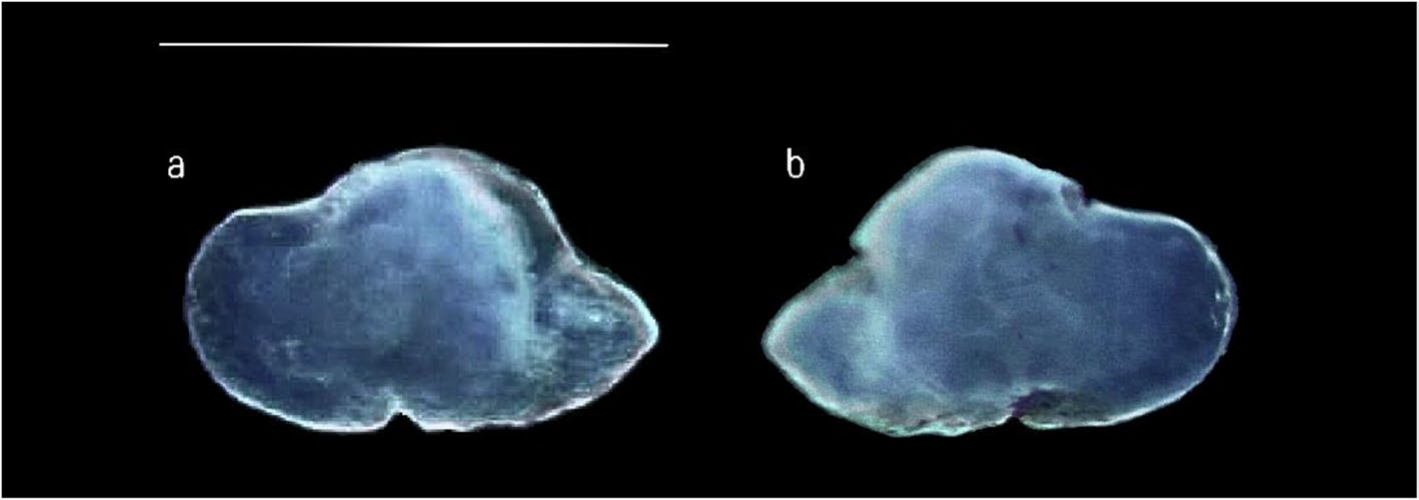
Stereoscope images of the dorsal (**a**) and ventral (**b**) sides of left *lapillus*. Scale bar: 0,5 cm

Following the terminology of Assis [57, 67], the studied species showed triangular *lapilli* with a non-clupeiform-type morphology, longer than wider (Fig. 6a, b). They showed a convex ventral face with a rounded, symmetrical junction between the ventral and dorsal faces. The dorsal face was generally smooth, with a not clearly lobed medial part. The *Extremum posterior* was lobed and smooth. At the same time, the *extremum anterior* was sharp and blunt pointed. *Prominentia marginalis* was little, triangular with a blunt apex, while *gibbus maculae* was large, with a slightly rough surface and an asymmetric, rounded outline, covering almost entirely the ventral otolith’s face. It did not cover completely *prominentia marginalis* on the ventral face, while from the dorsal one, it was clearly visible the *regio apicale gibbi*, almost entirely covering the dorsal outline of the *prominentia marginalis.* Anteriorly, the *sulcus lapillus* was clearly visible, sunken along the entire outline of the *gibbus maculae.*

In Table 4 they were reported the morphometric parameters calculated for the studied specimens for each side.

**Table 4:** *Lapilli* morphometric mean values, with standard deviation (s.d.), maximum (Max.), and minimum (Min.) values, for each investigated size class: maximum otolith length (OL, mm) and otolith width (OW, mm), otolith perimeter (OP, mm), otolith surface (OS, mm^2^), otolith length to the total fish length ratio (OL/TL), circularity (C = OP^2^/OS), rectangularity (Re = OS/[OL × OW]), ellipticity (E = OL–OW/OL + OW), aspect ratio (AR = OW/OL%), form factor (FF = 4πOS/OP^2^) and roundness (Ro = 4OS/πOL^2^)

	**R**			**L**		
	**Mean**	**s.d**	**Min.—Max**	**Mean**	**s.d**	**Min.—Max**
**OL**	0.53	0.06	0.37—0.62	0.52	0.04	0.43—0.6
**OW**	0.38	0.06	0.31—0.51	0.35	0.05	0.31—0.47
**OP**	1.61	0.2	1.29—1.95	1.56	0.15	1.3—1.84
**OS**	0.14	0.03	0.1—0.2	0.13	0.02	0.09—0.18
**OL/TL %**	0.2	0.04	0.13—0.24	0.2	0.04	0.14—0.26
**C**	18.32	0.65	17.28—19.63	18.7	0.69	17.77—20.11
**Re**	0.7	0.02	0.67—0.74	0.7	0.01	0.68—0.72
**E**	0.16	0.07	0.02—0.24	0.19	0.05	0.08—0.26
**AR**	0.73	0.11	0.61—0.95	0.68	0.07	0.58—0.86
**FF**	0.69	0.02	0.64—0.73	0.67	0.02	0.62—0.71
**Ro**	0.65	0.1	0.54—0.9	0.61	0.06	0.52—0.76

Eleven pairs of *Lapilli* were analyzed, and no significant variations of the morphometric parameters between the right and left sides were identified. It was not possible to perform comparative analyses between males and females as it was not possible to extract an equal number of *lapilli* from both sexes. For the same reason, the analysis was inaccurate for size class investigations.

**Fig. 7:**
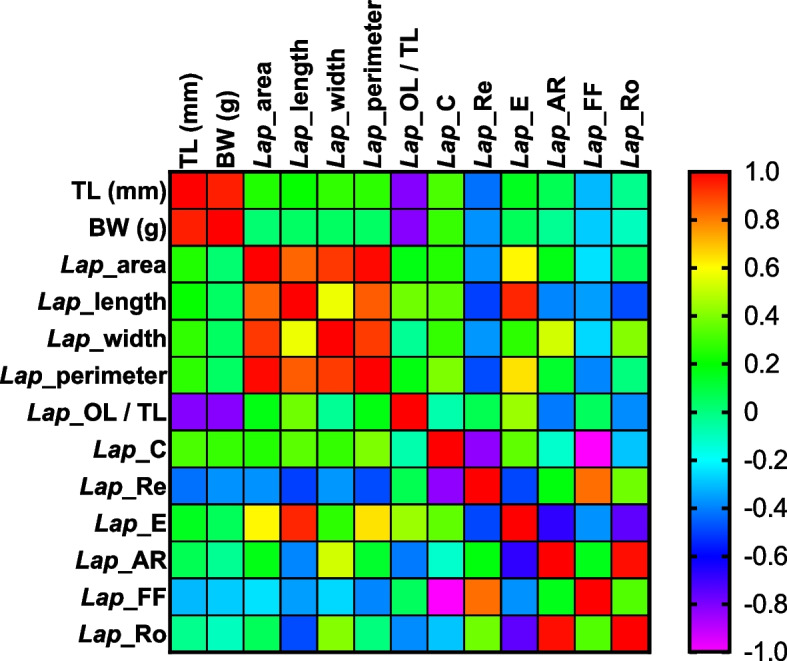
Pearson correlation matrix assessed between biometric specimens’ data (total fish length, TL, and body weight, BW) of the investigated species and morphometric *lapilli* parameters

Strong positive correlations were found between TL and BW of the specimens’ *vs* OL/TL (see Fig. 7).

**Fig. 8:**
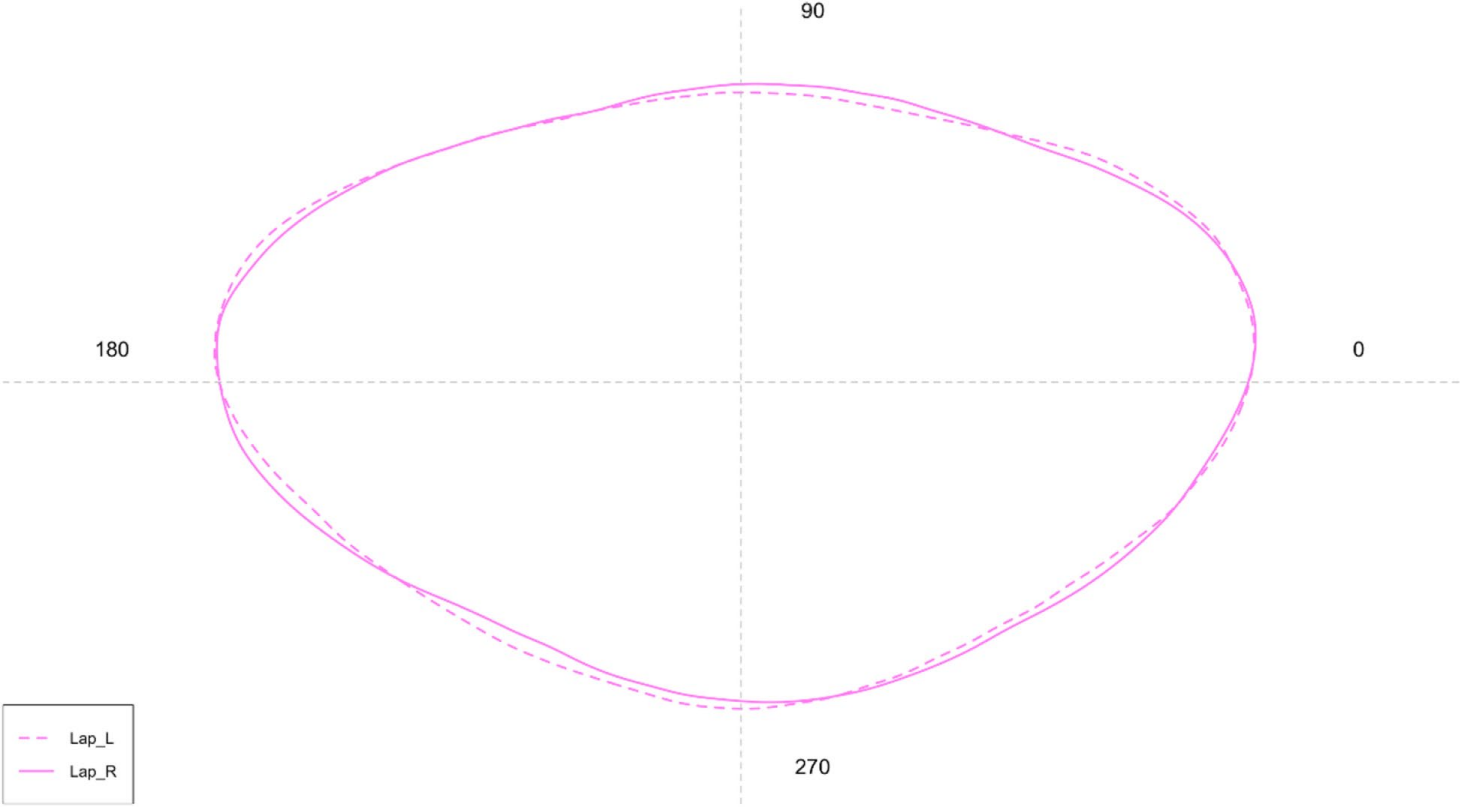
Mean shape of the left (L) and right (R) *lapilli* contours

Concerning the shape analysis, it showed a rhomboidal to oval mean contour of *lapilli* (Fig. 8)*.* The medial edge was regular, without the presence of distinct lobes. There was no incision between the *extremum posterior* and the lateral base of the *gibbus maculae*. The edge of the *extremum anterior* was also regular, not lobed. ANOVA reported no significant differences between the shape indices of left and right *lapilli.*

### Morphometric and Shape analysis of Asterisci

**Fig. 9:**
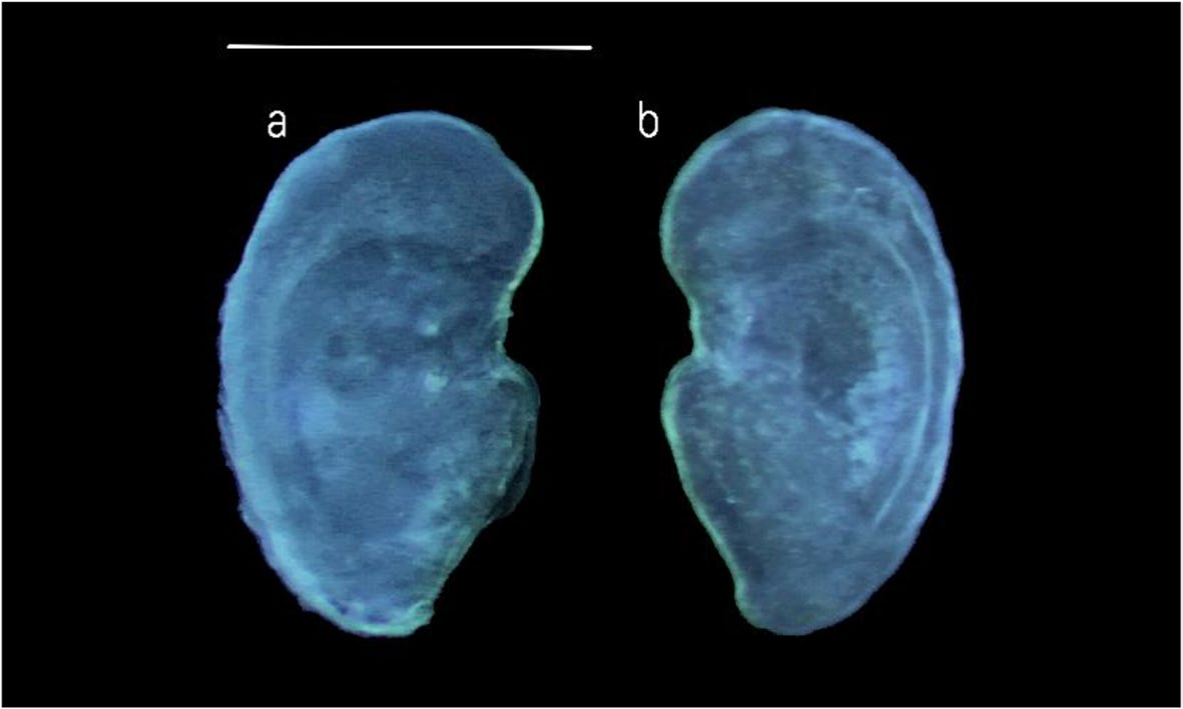
Stereoscope images of the external (**a**) and internal (**b**) faces of the right *asteriscus*. Scale bar: 0,5 cm

According to the terminology adopted by Assis [58, 67], the studied species showed vertical-type *asterisci* with a globular dorsal region and a pointed ventral one (Fig. 9a, b). The external face was dorsally flat and ventrally concave, with a slightly rough surface, while the inner face was markedly convex. *Lobi* were almost completely merged, not always clearly recognizable, with a furrow delimitating *lobus minor* and *lobus major.* This last was semi-oval, with a vertical axis much longer than the *lobus minor.* This was semi-circular, slightly anteriorly angled. *Rostrum* was short but always identifiable as a short, angled protuberance in the anterior otolith’s margin.

Conversely, *antirostrum* was large, globular, and very evident. It was placed in the anterior margin of the antero-dorsal part of the *lobus major*. *Excisura major* was wide, and not deep and the *fossa acustica* was antero-ventrally located, superficial, and very long. *Colliculumu* was very evident, covering a large part of the *fossa acustica.*

Table 5 reported the morphometric parameters calculated for the left and right otoliths of the studied specimens.

**Table 5:** *Asterisci* morphometric mean values, with standard deviation (s.d.), maximum (Max.), and minimum (Min.) values, for each investigated size class: maximum otolith length (OL, mm) and otolith width (OW, mm), otolith perimeter (OP, mm), otolith surface (OS mm^2^), otolith length to the total fish length ratio (OL/TL), circularity (C = OP^2^/OS), rectangularity (Re = OS/[OL × OW]), ellipticity (E = OL–OW/OL + OW), aspect ratio (AR = OW/OL%), form factor (FF = 4πOS/OP^2^) and roundness (Ro = 4OS/πOL^2^)

	**R**			**L**		
	**Mean**	**s.d**	**Min.—Max**	**Mean**	**s.d**	**Min.—Max**
**OL**	0.44	0.07	0.3—0.57	0.45	0.07	0.32—0.56
**OW**	0.74	0.09	0.58—0.9	0.71	0.08	0.54—0.85
**OP**	2.18	0.25	1.64—2.71	2.31	0.37	1.62—2.95
**OS**	0.24	0.06	0.13—0.38	0.24	0.06	0.14—0.35
**OL/TL %**	0.16	0.03	0.12—0.22	0.16	0.02	0.13—0.21
**C**	20.07	1.09	18.54—22.63	22.15	1.76	18.42—24.58
**Re**	0.73	0.03	0.69—0.77	0.75	0.02	0.71—0.78
**E (-)**	0.25	0.06	0.15—0.36	0.22	0.05	0.17—0.36
**AR**	1.69	0.22	1.38—2.13	1.59	0.17	1.4—2.1
**FF**	0.63	0.03	0.55—0.68	0.57	0.05	0.51—0.68
**Ro**	1.57	0.21	1.24—1.93	1.51	0.17	1.3—2.01

Fourteen pairs of *asterisci* were analyzed, and no significant variations of the morphometric parameters between the right and left sides were identified. It was not possible to perform comparative analyses between males and females as it was not possible to extract an equal number of otoliths from both sexes. For the same reason, the analysis was inaccurate for size class investigations.

**Fig. 10:**
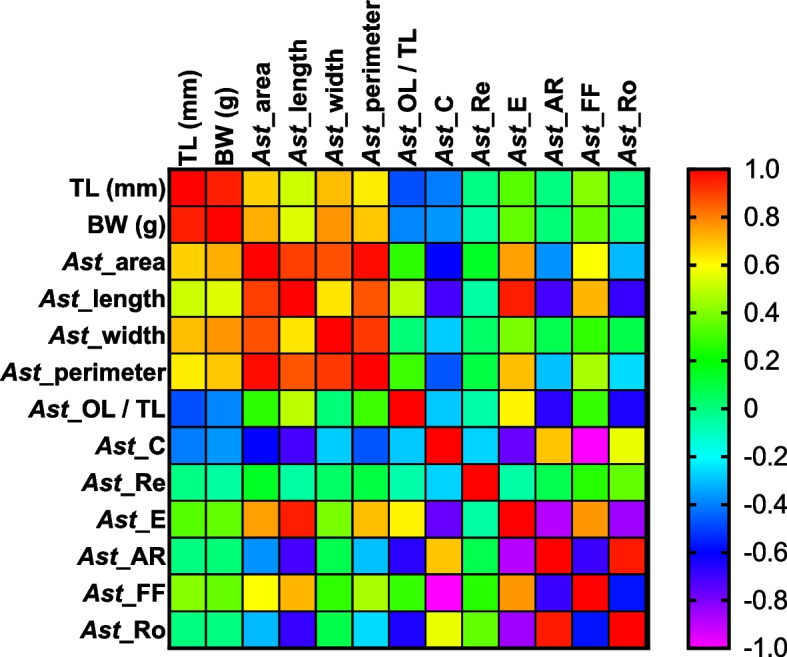
Pearson correlation matrix assessed between biometric specimens’ data (total fish length, TL, and body weight, BW) of the investigated species and morphometric *asterisci* parameters

Strong positive correlations were found between TL and BW of specimens vs. *Asterisci* area, length, width, and perimeter (see Fig. 10).

**Fig. 11:**
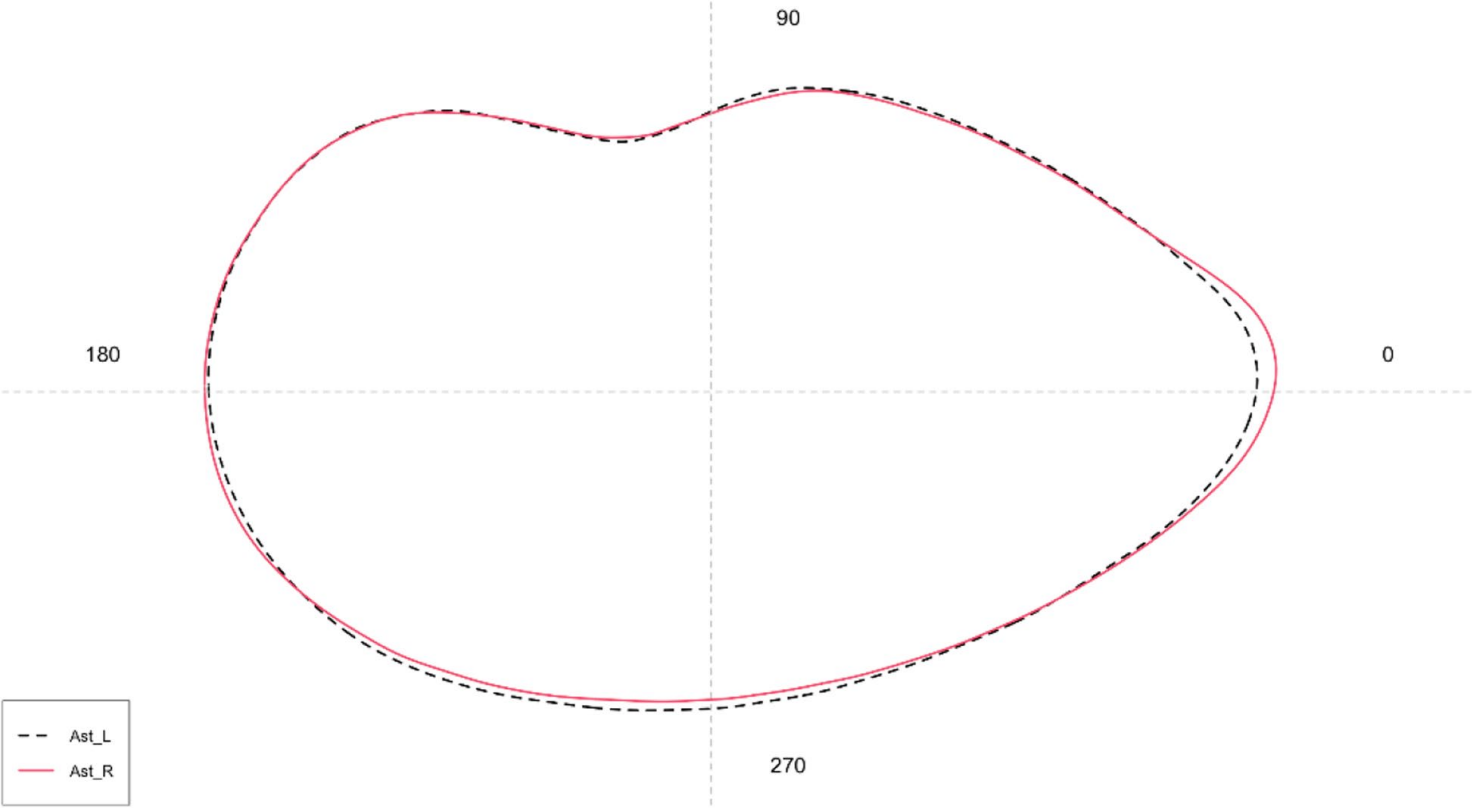
Mean shape of the left (L) and right (R) *asterisci* contours

Concerning the shape analysis, the mean *asterisci* contours showed an overall oval shape of the otoliths (Fig. 11). The dorsal region contour was globular, while the ventral was tapered and more pointed in the right *asterisci* than in the left ones. The *excisura major* was large, with a short and rounded *rostrum* and a large and globular *antirostrum.* ANOVA reported no significant differences between the shape indices of left and right *asterisci.*

## Discussion

The results from present study provided the first complete description of the three otoliths pairs of *B. belone* through morphometric, morphological and shape analysis, useful to understand their intra specific variability and how this could be influenced by the epipelagic habits of this species. It is important to explore the information stored inside these carbonate structures, able to provide reliable data on the life habits, large- or small-scale migrations, populations structure and habitats switches. Indeed, especially regarding the high migratory epipelagic species, it is often difficult to discriminate between populations, understanding their movements, life habit, and ecomorphological adaptation to epipelagic environment. These kinds of information are essential for conservation porpoises (for a correct fisheries management and resource conservation) and to improve the knowledge base on the ecological dynamics occurring in the pelagic domain. The studied species is an epipelagic species characterized by a migratory pattern, from the off-shore to the near-shore waters, related to spawning. Moreover, it shows a habitat switch during its growth, inhabiting the near-shore coastal environment, often close to freshwater streams, in the first part of the life, moving in the off-shore epipelagic environment during the adult life. The description of the mean otoliths shape and morphometry, and their intra specific variability, provided by results fill the gap present in literature (especially regarding utricular and lagenar otoliths) about the *B. belone* from the studied area, trying to analyze how its life habit can influence the three otoliths' pairs morphology, morphometry, and shape. Despite there is a lack of relevant information regarding the exact distribution of *B. belone* in the studied area (being the examined specimens purchased from the market), the local artisanal fisheries suppling the analyzed specimens operates in the Ionian Sea. For this reason, the studied group of individuals are considered belonging to the *B. belone* population inhabiting this geographical area FAO statistical division 37.2.2-Ionian Sea, as also highlighted by the differences detected in otoliths morphology and morphometry comparing data provided by results with literature from others geographical areas.

Concerning *sagittae,* morphological data showed an overall morphology in line with literature from the western and central Mediterranean Sea and the Black Sea [64, 66]. Shape indexes presented differences in aspect ratio and rectangularity, with markedly higher values in the studied population than that from the western and central Mediterranean Sea [66]. Differences were evident for both morphometric parameters (OL, OW, OP) and shape indexes (C, AR, Ro), also comparing data with those reported in the literature from the Aegean Sea [63, 80] and the Adriatic Sea [62]. These differences in shape indexes could suggest the reliability, in the studied species, of shape and overall contours for stock assessment and population discrimination, but, unfortunately, no literature data on shape analysis performed on *B. belone* from other geographical areas are available to confirm this. It is widely reported how morphometric and shape data on *sagittae* are reliable and useful to understand the population structure of migratory epipelagic species. This is the case of *Sardina pilchardus,* Walbaum, 1792. The population structure if this species in the Mediterranean Sea and Atlantic Ocean has been reconstructed successfully using otoliths shape descriptors, also elucidating the connectivity between Mediterranean and Oceanic individuals’ groups [81]. This is also the case of *Scomberomorus brasiliensis* Collette, Russo & Zavala-Camin, 1978, in which the *sagittae* phenotypic variation has been used as a stock structure’s indicator [69]. Otolith features’ variations between different geographical areas could be related to environmental and genetic differences between the areas and the populations, being otoliths under the dual regulation of environmental conditions and genetics [82, 83]. The main environmental factors influencing otolith morphometry, morphology, and shape are depth, temperature, salinity, diet composition, and food availability. These can alter somatic growth, fish metabolism, and otoliths features [49, 50, 84–86] under strong genetic control [81, 82, 87–89]. According to the literature, *B. belone* populations show substantial geographical variability in age at first maturity, growth rates, size ranges, sexes portions, age structures, and length distribution [12, 15, 17, 28]. This high geographical variability in population dynamics could reflect a strong sensitivity of the studied species to environmental conditions and fisheries activities. Indeed, many population descriptors, such as growth rates, abundance, length distribution, and age composition, are strongly affected by water masses, physiochemical and biological features, and fishing pressure [36, 90, 91]. Concerning feeding habits, studied species show a generally stable diet composition within the Mediterranean Sea and the Atlantic Ocean [18, 20, 21], with crustaceans as the main prey category and main differences related to crustaceans’ species contribution and minor preys’ items composition. The variability in diet composition, with the different environmental conditions experienced by the populations, and the inter-population genetic variability, could shape the variations between the different geographical areas in otolith features. Further analyses of the *sagittae* at inter-population level are required to understand and confirm the strong shape, morphological, and morphometrical differences between specimens of *B. belone* from different geographical areas. This is essential to assess the reliability of shape analysis for the stock assessment and population discrimination of the species, an essential tool to improve its conservation in the entire Mediterranean basin.

As highlighted by statistical analyses on shape data, the mean sagittal contour of the specimens from the Ionian Sea was not affected by bias related to intra-specific differences between otoliths’ sides and sexes. This, indeed, is one of the main factors that could affect the accuracy of shape analysis for stock assessment, being this strongly altered by choice of *sagittae* from one side rather than the other, or from one sex rather than the other, in the presence of directional bilateral asymmetry or sexual asymmetry [92–94]. Statistical analysis has detected significant differences between the three size classes in morphometry and shape. This strong relation between *sagittae* morphometry and morphology and specimens’ biometry (total length and body weight); Pearson correlation results also confirmed it. This finding was in line with literature from the Adriatic and Aegean Seas [62, 63], which reported a strong correlation between total fish length and otolith morphometry for *B. belone*, the strongest among several investigated pelagic species, essential for the back calculation of fish length from the otolith one. The high variability in shape between the *sagittae* belonging to the three investigated size classes could be related to the life habits of the species. For instance, according to Dorman [18], smaller specimens of *B. belone* seem to feed more frequently at nearshore surface waters than larger ones, resulting in differences in diet composition. In addition to the contribution of different prey items, the variations in environmental conditions are experienced by smaller and adult specimens, being nearshore water characterized by different physiochemical properties and oceanographic features than the offshore ones. This environmental variability could allow the differences detected between size classes in sagittal shape, but further analysis of the life habits of the studied species in the Ionian Sea are required to confirm this thesis. Moreover, other studies with a widest as possible number of specimens for each size classes and sex are required to confirm the information regarding the intra specific variability in otoliths’ shape and morphology provided by results. Indeed, the number of specimens considered in present study is not enough for making some solid conclusions regarding the development of otoliths during the life cycle of the studied species, or their sexual dimorphism.

Concerning *lapilli,* results showed a different morphology than those reported literature from the Atlantic coast of Portugal [57, 67]. As stated by Assis, the variability was mainly related to the general otolith’s shape and the *gibbus maculae* dimensions. Indeed, specimens from the Ionian Sea showed a more oval and rhomboidal than triangular shape, with less bulky *gibbus maculae* than those shown by specimens from the Portugal coast. These differences between shape and morphological results and literature data could indicate an inter population variability of *lapilli*, not investigated at all in literature. Unfortunately, no references with comparable data are present in literature to assess the morphometrical distance of *lapilli* at inter-population level*.* The detected differences with the literature in morphology and shape confirm the need to improve the knowledge base on the intra-population variability of these otoliths, which nowadays are deeply underestimated [59].

Concerning *asterisci,* results showed a morphology in line with that reported in the literature from the Atlantic Portuguese coast [58]. Some differences were detected regarding the general shape, more oblong in the studied population and the *rostrum,* less evident in the studied population, than those from the literature. As stated above, for *lapilli,* these differences could indicate a variability at the inter-population level, until now not investigated at all in *asterisci*. The absence of literature on morphometrical data made it impossible to compare the morphometry of *B. belone asterisci* from the Ionian Sea with that from other geographical areas. As highlighted by the detected differences in morphology and shape with literature data, it is essential to improve the knowledge base on the intra-population variability of these otoliths, until now deeply underestimated [59].

At the intra-population level, statistical analyses on *lapilli* and *asterisci* did not show significant differences between left and right otoliths. Concerning size classes, especially *asterisci,* showed a strong correlation between otoliths morphometries (e.g., OS, OL, OW, and OP) and specimen biometrics (TL and BW), as stated by Pearson correlation results. But, as stated above, further analysis investigating a widest as possible number of specimens for each size classes are required to confirm the information regarding the intra specific variability in otoliths’ shape and morphology provided by results. The correlation between morphometries of *asterisci* and *lapilli,* and total fish length and weight could introduce a substantial variability between small and large individuals in the studied species from the Ionian Sea. This may be related to the life habit differences between life stages of *B. belone*, stated above, to discuss the size class differences detected in *sagittae.* It was widely stated that *saccule* and *lagena* are mainly involved in the perception of sound, while the *utricle* seems to have an important role in vestibular sense [59, 95, 96].

For this reason, *sagittae, asterisci,* and their end organs could be influenced by the same selective forces, explaining the more enhanced correlation of morphometries to fish total length and body weight shown by these otoliths than *lapilli.* These last have always been considered the most conservative of the three [57], despite Schulz-Mirbach et al. [59] having shown their variability at the inter-specific level, assessing differences also in the development of vestibular sense. Further analysis on *lapilli* and *asterisci* from different populations of the studied species, and with a vast number of samples, are required to confirm the reliability of shape, morphology, and morphometry for population discrimination and to assess the variability at intra-population between sexes and size classes.

## Conclusion

Results provided the first accurate description of the three otoliths pairs of the studied species from the Ionian Sea, with the first-ever description of *lapilli* and *asterisci* from the Mediterranean Sea.

These data have confirmed the heterogeneity of sagittal morphology and morphometry between the present paper and literature, also highlighting the presence of differences in mean contour and morphometry between size classes. The absence of directional bilateral asymmetry and sexual asymmetry lets us hope for a reliable and straightforward application of shape analysis for the stock assessment on this species, which is essential for its conservation and correct management of its commercial exploitation.

Concerning *lapilli* and *asterisci,* results have confirmed the need to deepen the knowledge of these two otoliths pairs, not studied at all in the Mediterranean teleost species. They showed differences in morphometry and shape with literature data, which could indicate an intra-specific variability between specimens belonging to different populations. Improving the knowledge base on this is essential to understanding how different environments can influence the inner ear development and morphology and how the vestibular and hearing senses change between species and populations according to their life habits and adaptation to environments. Moreover, for both otolith pairs, it was not possible to investigate the variability between size classes and sexes in the studied population for lack of samples. Further analyses on a broader number of lagenar and utricular otoliths are required to analyze their variability between size classes and sexes.

Future research on the three otoliths pairs of the studied species shall investigate the reliability of shape analysis for stock assessment and population discrimination, adding data on genetics, somatic growth dynamics, and feeding habits on specimens from different geographical areas. This will be essential to find direct correlations to elucidate the dynamics influencing the inter-population differences in *sagittae, lapilli,* and *asterisci,* improving the information about the connections between inner ears, species and population genetics, and environment. This is the base of the phenotypic plasticity and the ecomorphological adaptation of teleost species, allowing the shape differences between *sagittae,* so crucial for stock assessments and conservation porpoises. Moreover, by deepening the knowledge base on the intra and inter-specific variability of lagenar and utricular otoliths, elucidating their variations related to genetic and environmental conditions, it will be possible to improve the information about teleost inner ear functioning and eco-morphology, opening new ways for species population discrimination.

### Supplementary Information

**Supplementary Material 1.**

**Supplementary Material 2.**

## Data Availability

All data generated or analysed during this study are included in this published article [and its supplementary information files].
