# Supplementary material for: Intra-population variability of the saccular, utricular and lagenar otoliths of the garfish Belone belone (Linnaeus, 1760) from South-Western Ionian Sea (Central Mediterranean Sea)
Source: BMC Ecol Evol. 2024 Mar 11;24:31. doi: 10.1186/s12862-024-02219-0 (PMC10926657; doi:10.1186/s12862-024-02219-0)
Supplement: Supplementary file 1 — Supplementary Material 1. [file 12862_2024_2219_MOESM1_ESM.docx]

a)


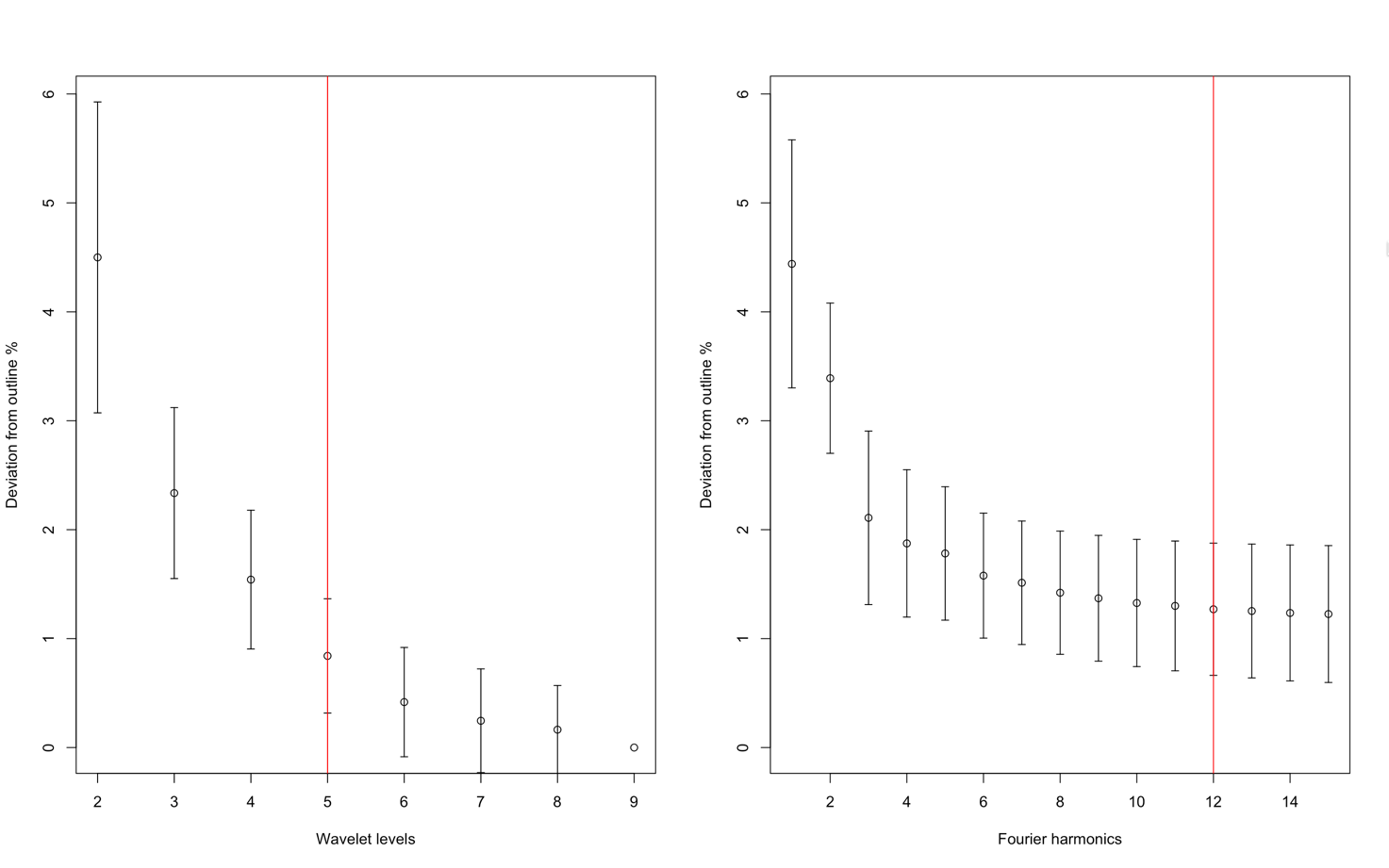

b)


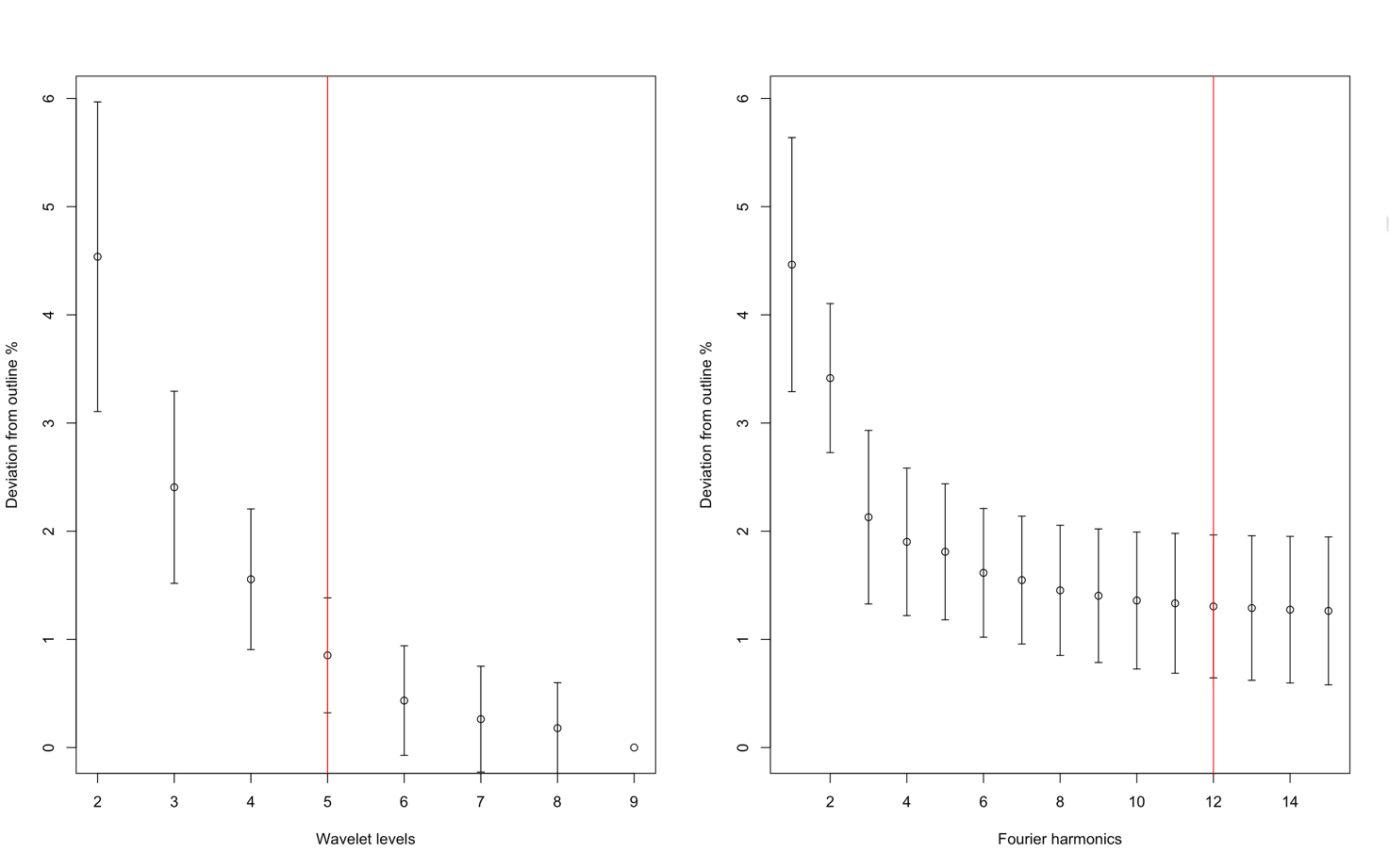


Figure S1a. Quality of the Wavelet and Fourier outline reconstruction of *sagittae* extracted from male and female specimens of *B. belone*. The red lines indicate the level of Wavelet and number of Fourier harmonics needed for a 98.5% accuracy of the remodelling.

Figure S1b. Quality of the Wavelet and Fourier outline reconstruction of *sagittae* extracted from specimens of *B. belone* belonging to different size classes. The red lines indicate the level of Wavelet and number of Fourier harmonics needed for a 98.5% accuracy of the remodelling.
