# Supplementary material for: Intra-population variability of the saccular, utricular and lagenar otoliths of the garfish Belone belone (Linnaeus, 1760) from South-Western Ionian Sea (Central Mediterranean Sea)
Source: BMC Ecol Evol. 2024 Mar 11;24:31. doi: 10.1186/s12862-024-02219-0 (PMC10926657; doi:10.1186/s12862-024-02219-0)
Supplement: Supplementary file 2 — Supplementary Material 2. [file 12862_2024_2219_MOESM2_ESM.docx]

a)


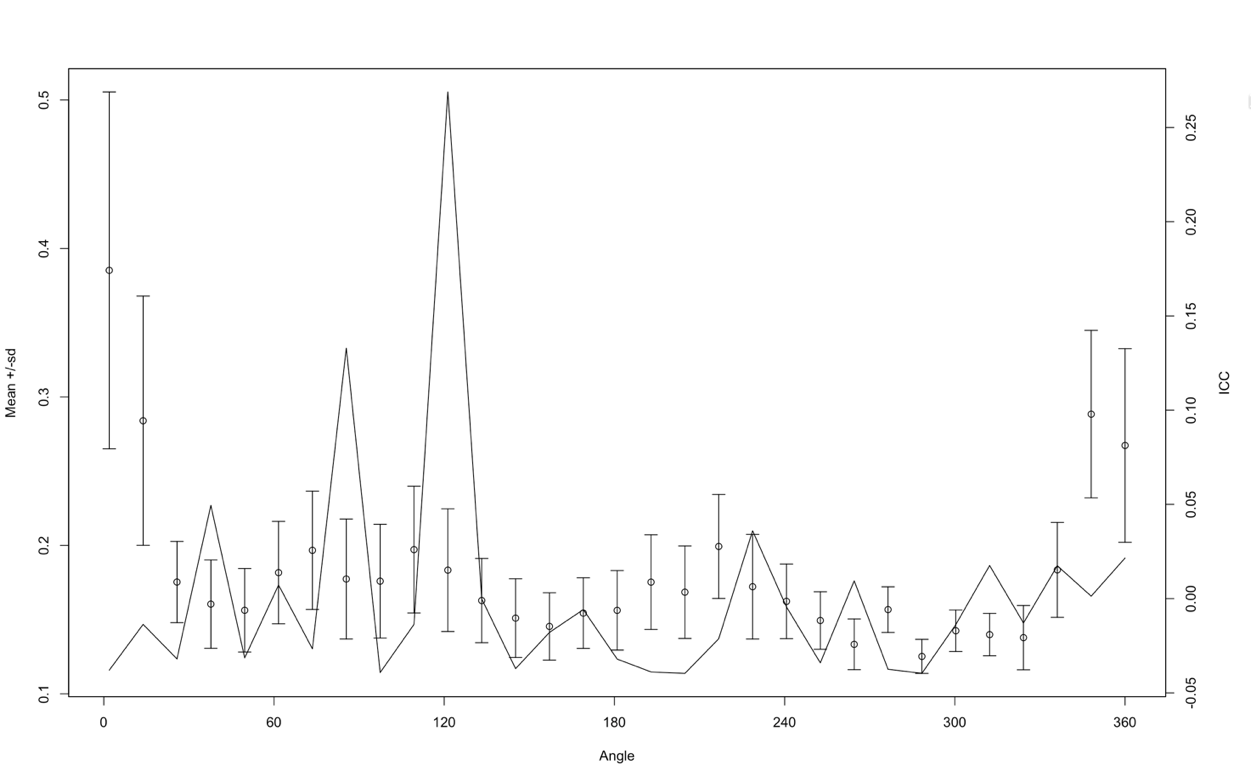


b)


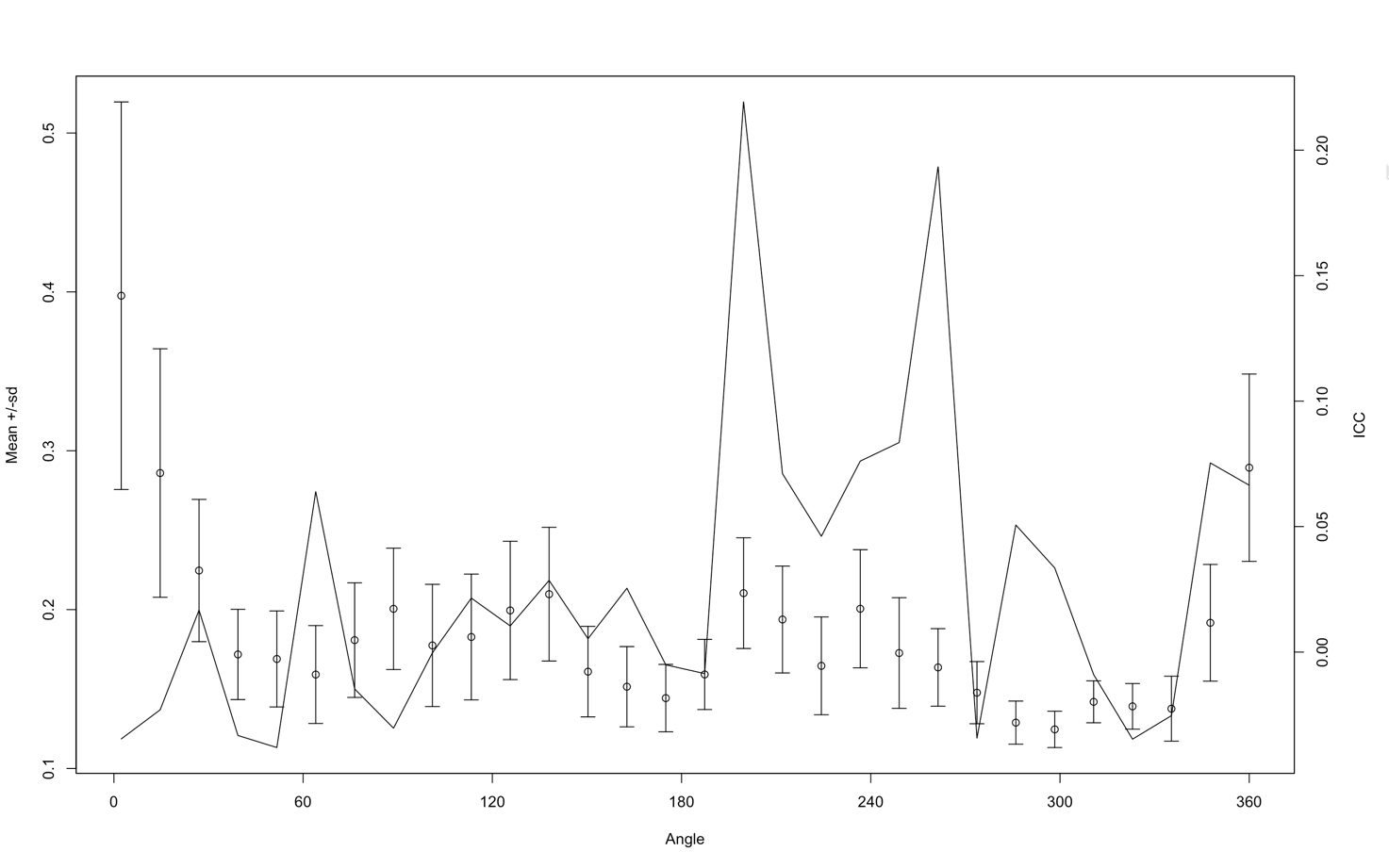


Figure S2a. Mean and standard deviation (SD) of the Wavelet coefficients for all *sagittae* combined and the proportion of variance between male and female individuals of *B. belone* (black line). The angles in degrees (°) indicated on the horizontal axis are based on the polar coordinates of the otolith mean shape plot (FIGURE 4B). The centroid of the otolith is the central point of the polar coordinates.

Figure S2b. Mean and standard deviation (SD) of the Wavelet coefficients for all *sagittae* combined and the proportion of variance among the three size classes (black line). The angles in degrees (°) indicated on the horizontal axis are based on the polar coordinates of the otolith mean shape plot (FIGURE 4A). The centroid of the otolith is the central point of the polar coordinates.
